# Intracranial Arterial Calcifications: Potential Biomarkers of Stroke Risk and Outcome

**DOI:** 10.3389/fneur.2022.900579

**Published:** 2022-09-01

**Authors:** Gianna M. Fote, Sophia Raefsky, Kelton Mock, Amit Chaudhari, Mohammad Shafie, Wengui Yu

**Affiliations:** ^1^School of Medicine, University of California, Irvine, Irvine, CA, United States; ^2^Department of Neurosciences, University of California, San Diego, La Jolla, CA, United States; ^3^Department of Neurology, University of California, Irvine, Irvine, CA, United States

**Keywords:** stroke, calcification, atherosclerosis, imaging, biomarker

## Abstract

Intracranial artery calcifications (IAC), a common and easily identifiable finding on computed tomorgraphy angiography (CTA), has gained recognition as a possible risk factor for ischemic stroke. While atherosclerosis of intracranial arteries is believed to be a mechanism that commonly contributes to ischemic stroke, and coronary artery calcification is well-established as a predictor of both myocardial infarction (MI) and ischemic stroke risk, IAC is not currently used as a prognostic tool for stroke risk or recurrence. This review examines the pathophysiology and prevalence of IAC, and current evidence suggesting that IAC may be a useful tool for prediction of stroke incidence, recurrence, and response to acute ischemic stroke therapy.

## Introduction

Intracranial artery calcifications (IAC) are a common finding on computed tomography (CT) or CT angiography. In recent years, IAC has been recognized as a possible risk factor for ischemic stroke (1–3). Arterial calcification generally occurs as a diffuse process involving the medial layer or as localized plaques involving the intimal layer, however, these patterns have not been well-characterized in the context of intracranial arteries (4). Intimal calcification is commonly observed on CTA and more frequently associated with plaque and luminal stenosis in intracranial arteries, suggesting a potential contribution to a heightened risk of ischemic stroke (5–7). Despite this potentially increased risk, currently, no widely accepted clinical or radiographic tools exist to investigate the influence of intracranial artery calcification on the risk of ischemic stroke. This review focuses on the current understanding of intracranial artery calcification, its implications as a risk factor for ischemic stroke as well as predictive tools for its stratification and management.

## Methods

This is a narrative review of literature on intracranial calcifications and stroke. On March 2020, the National Library of Medicine's Pubmed database for searched with the following string: “intracranial” AND “calcification” AND “stroke”. Results were filtered by (1) English language, (2) availability of full text, (3) human study population, and (4) publication date within the last 20 years (i.e., from January 2000 onwards). The search yielded at total of 192 articles. Abstracts for all articles were independently reviewed by three different authors on the paper. Articles were selected for inclusion based on (1) relevance to the topic and (2) presentation of original clinical data. Articles that were irrelevant, had incomplete data or were theoretical/analytical without presentation of original clinical data were excluded. Articles that met inclusion criteria by any one reviewer, even if it did not by the other two reviewers, were still included in the analysis. It is of note that for the most part all three reviewers were in consensus as to the inclusion or exclusion of individual articles. A total of 26 articles were selected, and a comprehensive analysis is presented here. All research was conducted in accordance with the ethical and scientific guidelines put forth by the Institutional Review Board at the University of California Irvine.

## Pathophysiology: Role of Structure and Function

Intracranial arteries have some important structural differences from their more well-studied coronary counterparts. These differences include a more dense internal elastic lamina, thinner media and adventitia with less elastic fibers, less vasa vasorum, and lack of an external elastic lamina in intracranial arteries. In a 2017 review, Yang et al. propose that the paucity of elastic fiber in intracranial arteries may constitute a protective feature, as elastic fibers are thought to be more prone to inflammation, fibroproliferative response, and atherosclerotic damage (4). On the other hand, it has also been suggested that a decreased expression of anti-inflammatory molecular mediators in the intracranial artery microenvironment may actually cause increased susceptibility to plaque instability and inflammatory changes in comparison to extracranial arteries (8). Thus, while a comprehensive comparison between intracranial and extracranial artery microstructure has yet to be conducted, it is likely that calcification and atherosclerosis look very different in intra- vs. extracranial settings.

### Arterial Calcification Patterns

**Figure 1:**
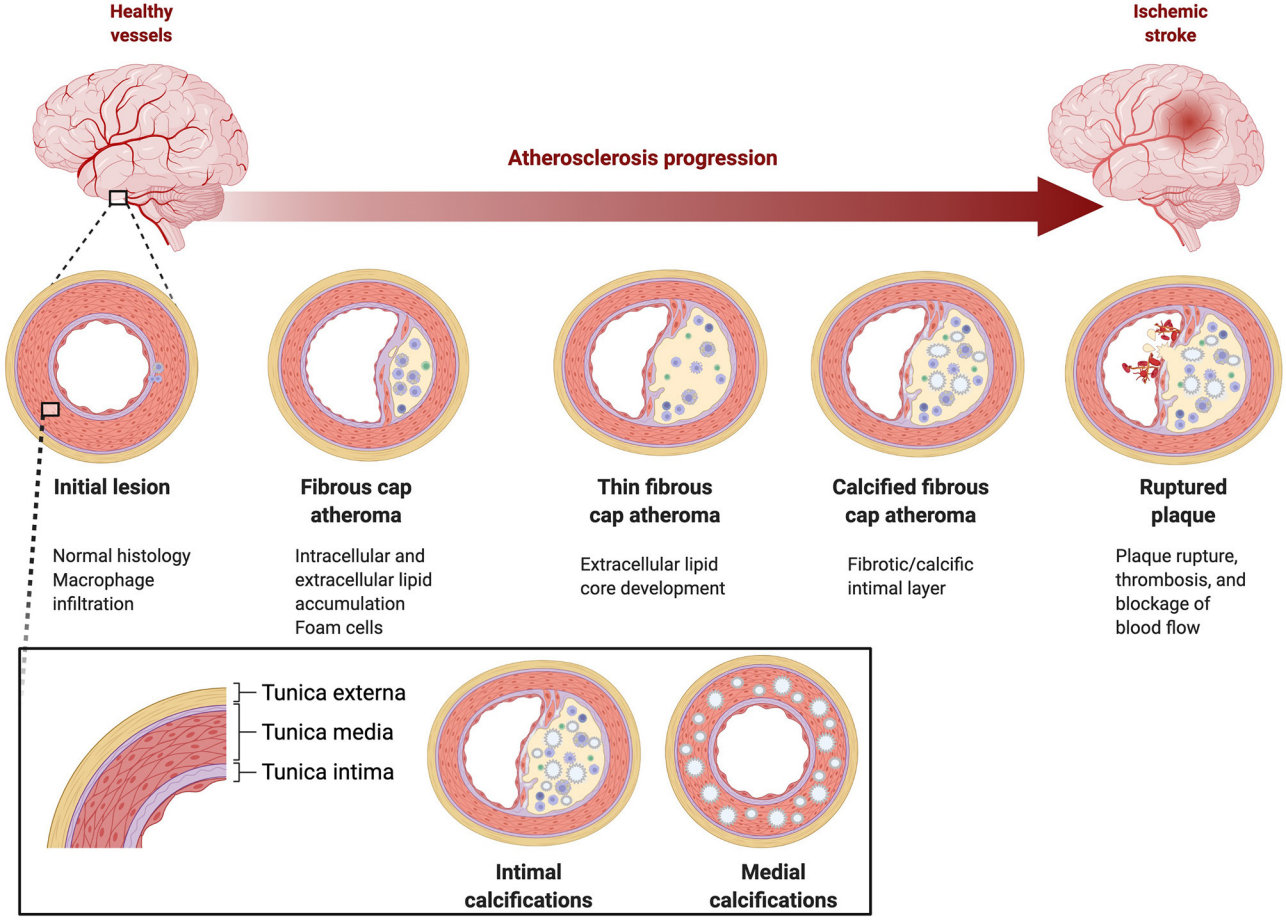
Pathophysiology of intracranial atherosclerosis. Large intracranial vessels are muscular arteries with three wall layers, tunica externa, tunica media, and tunica intima.

Calcification may occur in the muscular medial layer or the endothelium of the intimal layer of the artery wall (see Figure 1). Age, pulse pressure, and family history are risk factors for both types of calcifications, whereas smoking and hypertension are risk factors for intimal calcification and diabetes and chronic kidney disease are risk factors for medial calcification (9, 10). Intimal calcification is considered a marker of atherosclerosis. Atherosclerosis progresses from endothelial damage to the formation of fibrous plaques with a core of lipid, cholesterol, and cellular debris under a cap (11). For reasons that are still not well understood, atherosclerotic lesions become calcified *via* a molecular mechanism that is similar to bone formation (11). While some research findings have suggested that the process of calcification has a stabilizing effect on associated plaque, others suggest that this relative structural stability may trade off with a higher overall plaque burden, and potentially plaque instability, in heavily calcified arteries (12–19).

While atherosclerosis of the intima is presumed to contribute to stroke the clinical significance of medial calcification is unknown, thus, a challenge in scoring IAC lesions is differentiating between intimal and medial calcifications. Histological examination is still the most reliable way to differentiate between intimal and medial calcification, however a scoring system, Kockelkoren scoring, is based on CT imaging and incorporates circularity, thickness, and morphology to differentiate medial and intimal calcification in the intracranial internal carotid artery (20). Kockelkoren scoring has been used to evaluate differential effectiveness of stroke interventions (tPA or endovascular thrombectomy) (21, 22).

One cross-sectional study verified the validity of CT assessment of IAC by obtaining a CT scan prior to autopsy and histological evaluation (23). The calcification patterns seen on CT were correlated with the histological findings. Sixty seven percent of calcifications detected were in the intima, and the majority of calcification was observed in the vertebral artery (51%). All of the intimal calcifications were in progressive atherosclerotic lesions, and arteries with intimal calcification had more severe luminal stenosis than those without (23). In contrast, a large cross-sectional study of acute stroke patients by Vos et al. assessed localization of IAC (intimal vs. medial) by non-contrast CT (9), and found that dominant intimal calcification was present in 30.9% of subjects and dominant medial calcification in 46.9% of subjects, with no calcification in 10.5%. A histological study by Vos et al. found that calcification of the intracranial ICA was predominantly found in the internal elastic lamina, and was not related to the occurrence of intimal atherosclerotic lesions (24). The arterial wall layer where calcification depend on the specific intracranial artery being examined and the method by which calcification is assessed. Future studies are needed to determine whether the majority of vertebral artery calcifications are associated with atherosclerosis whereas the ICA calcifications are not, and whether this affects the risk of stroke for these vessels when other variables are controlled for.

## Prevalence of IAC

### Ethnicity and Prevalence of Intracranial Atherosclerosis

Among the limited available studies, some differences based on race and ethnicity have been observed. Although Japanese patients tend to have less atherosclerotic burden of the aorta, intracerebral atherosclerosis was more severe and occurred earlier in a study of Japanese patients than previously reported studies of Caucasians (25). Compared with studies of Caucasian and Japanese populations, Chinese patients have higher prevalence of cerebral atherosclerosis (26–28). In direct comparisons, Africans (29), African Americans (30), patients of African heritage in Europe (31), and a combined African American and Hispanic population (32) had more atherosclerotic lesions of intracranial arteries than Caucasians. Future studies directly comparing calcification of atherosclerotic lesions across populations of different ethnicities are needed.

Stroke patients have a high degree of IAC on imaging. Acute ischemic stroke patients in Nigeria and China were found to have high IAC burden (93 and 72% of subjects respectively) (29, 33). Calcification was mostly located in proximal inflow arteries in both studies.

### Anatomical Distribution of IAC

The anatomical burden of IAC has been determined by a handful of X-Ray and CT studies to lie most prominently in the anterior circulation involving intracranial internal carotid arteries, followed by the vertebral arteries and the basilar artery, although there has been some variation across studies (Table 1) (23, 29, 33, 35, 37).

**Table 1:** Anatomic distribution of intracranial artery calcification.

**% of IAC-positive scans with calcification of the indicated vessel**	**Yang et al. (34)** **(*N* = 32)**	**Chen et al. (33)** **(*N* = 200)**	**Olatunji et al. (29)** **(*N* = 130)**	**Chen et al. (2)** **(*N* = 175)**	**Chen et al. (35)** **(*N* = 340)**	**Bugnicourt et al. (36)** **(*N* = 259)**
ICA		64.8	86.1	80.4	66.9	72.9
MCA	35	6.3	2.4	7.3	8.8	4.4
ACA		1.7	1.0	3.6	2.0	*N/A*
VA	51	30.2	9.3	35.6	26.7	37.3
BA	14	19.5	1.2	4.5	5.1	3.5

## Ischemic Stroke Risk Prediction

### Clinical Predictive Tools

The majority of strokes in the United States are ischemic (87%) and atherothrombotic disease is the leading cause of ischemic stroke (38). Modifiable risk factors contributing to development of atherothrombotic disease including hypertension, hypercholesterolemia, atrial fibrillation, tobacco use, diabetes mellitus, physical inactivity, and obstructive sleep apnea (33, 39, 40). The most commonly used clinical predictive tools are the atherosclerotic cardiovascular disease (ASCVD), the CHA_2_DS_2_-VASc score in the context of atrial fibrillation, and the ABCD2 score for stroke risk following TIA. Currently there are no widely used clinical tools or algorithms which incorporate atherosclerosis or IAC to predict occurrence of ischemic stroke. Most patients who undergo neuroimaging do so in the context of a suspected stroke or TIA. Therefore, the distinction of risk factors associated with IAC from those attributed to ischemic stroke may prove challenging. The major risk factors for IAC include age, male sex, hypertension, diabetes, hypercholesterolemia, ischemic stroke, and chronic kidney disease, all of which are also considered major risk factors for stroke (1, 29).

### Scoring Methods for IAC and Ischemic Stroke Risk

The evaluation of IAC on head CT has largely followed principles established in the CAC literature, where coronary artery calcification (CAC) has been well-established as a predictor of both MI and ischemic stroke risk (39). Typically areas of high attenuation in arterial walls are interpreted as intra-arterial calcifications; in support of this methodology, a study by Denzel et al. showed good concordance between calcifications detected on CT and histologic analysis of carotid artery samples (41).

There are a variety of different techniques to calculate CAC, including both manual and automated techniques (42). The most widely used method is the Agatston method, whereby a specific set of serial transaxial cardiac CT images are read manually to generate a score based on the density and area of all coronary artery lesions identified throughout the series (43). CAC scoring by the traditional Agatston method has demonstrated consistent value in predicting cardiovascular events, including the ability to predict 10-year coronary heart disease risk with greater accuracy than diabetes, stroke, and other traditional risk factors (34, 44–59). Agatston scoring is currently used as a screen for clinically silent coronary ischemia (60). The Agatston scoring method was ubiquitous among the articles we reviewed (Table 2). However, the predictive value of this method for stroke outcomes is not clear: two of the included studies found no association of Agatston score with stroke risk, while three found some association with stroke incidence or recurrence. Limited emerging evidence suggests that new, alternative scoring methods may have increased predictive power for determining stroke outcomes over the Agatston method. The use of density and volume measurements, for example, showed improved prediction of CVD events in a subset of MESA (Multi-Ethnic Study of Atherosclerosis) participants when compared to traditional Agatston scoring (73). Modified methods similar to Agatston have been described with improved reproducibility (74).

**Table 2:** Intracranial arterial calcification and stroke risk.

**References**	**Study type**	**Patients**	**Scoring method**	**Stroke association with IAC & Conclusions**
Sohn et al. (5)	Retrospective, Case-Control	57 consecutive ischemic stroke patients	Yes/No IAC	Yes, large artery atherosclerotic or lacunar stroke subtypes
Taoka et al. (61)	Cross-Sectional	Consecutive patients older than 50 years	Agatston score	No significant association with stroke risk (2 years later)
Chen et al. (35)	Cross-Sectional	484 patients referred for brain CT	Yes/No IAC	
Chen et al. (2)	Retrospective, Case-Control	Ischemic stroke patients (175) and non-ischemic controls (182)	Yes/No IAC	Ischemic stroke association, IAC is an independent risk factor
Erbay et al. (62)	Retrospective	65 patients with CT and MRI	IAC vertebrobasilar and ICAC rated 1–4	Acute small-vessel infarcts were significantly associated with high ICAC
de Weert et al. (63)	Retrospective, Case-Control	406 patients with amaurosis fugax, TIA, or ischemic stroke	Yes/No IAC (modified Agatston)	No significant association with stroke laterality or stroke type
Bugnicourt et al. (36)	Retrospective, Case-Control	Consecutive ischemic stroke patients (379) and non-stroke neurological patients (171)	Semiquantitiative	Ischemic stroke association, no significant association with length of hospital stay on multivariate analysis
Bugnicourt et al. (64)	Prospective, Cross-Sectional	All ischemic stroke patients admitted to a single stroke unit over 1 year (302)	Semiquantitiative	Significant difference in the rates of death and vascular events between the highest and lowest IAC score groups
Power et al. (65)	Retrospective, Case-Control	Hemodialysis patients receiving CT scan for any neurological condition (529)	Semiquantitative	Greater IAC severity was an independent predictor of ischemic stroke. High-grade IAC was significantly associated with a higher age-adjusted risk of death
Bos et al. (3)	Prospective cohort study	2,323 stroke-free people followed for 6–9 years	ICAC volume	Large ICAC volume related to higher risk of stroke
Hussein et al. (66)	Retrospective	172 patients with subarachnoid hemorrhage	Volume and density of ICAC lesions	Highest tertile of calcification independently associated with less vasospasm
Lee et al. (67)	Prospective	1,017 patients with acute ischemic stroke and TIA	IAC categories: no IAC, mild IAC, severe IAC	Severe IAC was significantly associated with early progression/recurrence of stroke and poorer functional outcome after 3 months.
Wu et al. (42)	cross-sectional	68 patients from previous clinical study, consecutive ischemic stroke patients with MCA territory infarctions	semi automatic custom-made program	On ipsilateral iICA the presence of MES was more frequent in the higher calcification group
Kamel et al. (68)	prospective	55 patients with ICA territory cerebral infarction. Stroke of undetermined etiology (35) and cardioembolic stroke (21)	Agatston method	I stroke of undetermined etiology but not cardioembolic stroke, greater calcification in the ICA ipsilateral to infarction
Vos et al. (9)	cross-sectional	1,132 patients from Dutch acute stroke cohort	Semi-quantitative	Dominant intimal ca was present in 30.9% and medial 46.9% of subjects, 10.5% no calcification seen.
Gocmen et al. (22)	retrospective	91 consecutive acute anterior circulation stroke patients treated with IV tPA	Kockelkoren method	Carotid intimal calcification associated with higher tPA effectiveness, carotid medial calcification associated with risk of ICH with tPA
Compagne et al. (21)	Prospective	344 patients with acute ischemic stroke, MR CLEAN trial, randomized patients between EVT or no EVT (medical, tPA allowed)	Kockelkoren method	Benefit of EVT in AIS is greater in patients with medial calcification pattern than intimal Ca pattern. No association between ICAC volume and functional outcome
Olatinji et al. (2018)	cross-sectional	130 consecutive acute ischemic stroke patients	evaluated in bone window on vitrea software for extent, thickness, and length of calcifications	IAC in 93.1% of patients. Burden of IAC: mild (17.4%, moderate (52.1%), severe (30.6%)
Yang et al. (34)	observational	32 consecutive autopsy cases who died age >45 in hospital in Hong Kong	Bilateral arteries were extracted for each subject and stained with H&E and Victoria blue, Ca pattern identified on CT were correlated with histology	Visible calcifications detected in 39% segments. Intimal ca are related with progressive atherosclerotic lesions
Kong et al. (69)	prospective	156 consecutive TIA patients	Siemens Syngo. *via* calcium scoring system for post processing images	Higher CT calcium score was significantly associated with recurrent TIA/AIS.
Chen et al. (33)	prospective	276 consecutive patients with TIA or acute ischemic stroke	CT Agatston method	IAC present in 72.46% of patients. IAC is highly correlated with WMH, lacunae, and CMBs on MRI
Magdič et al. (79)	Case-control	448 consecutive stroke patients	Hyperdense area exceeding >90 Hounsfield units	Vertebrobasilar artery calcification associated with higher risk of recurrent stroke and vascular events.
Wu et al. (70)	Prospective	Prosepctive stroke registry 694 patients	Agatston method	Higher IAC Agatston score was associated with higher risk of recurrent stroke, post-stroke mortality, and small vessel occlusive stroke.
Yu et al. (20)	Retrospective	242 patients with acute non-cardiogenic ischemic stroke who received IV thrombolysis	Calcification volume	Arterial calcification volume on the lesion side is associated with hemorrhagic transformation after thrombolysis. The poorer prognosis group had more calcified vessels
Kauw et al. (71)	Prospective multicenter cohort study	982 patients with acute ischemic stroke	Yes/no ICAC, medial vs. intimal	IV thrombolysis was associated with favorable clinical outcomes and successful recanalization in patients with medial but not intimal ICAC
Bos et al. (72)	Prospective	1,349 people from population-based Rotterdam study	Yes/no ICAC	Calcification was not associated with stroke

Of the other scoring methods we encountered in our review, six studies used a simple binary yes/no to indicate whether calcification was observed. Six additional studies used semiquantitative methods involving categories with some degree of subjective interpretation. For example, in Power et al. (65), calicification of the carotid siphon was scored as Grade 0—absent, Grade 1—thin, discontinuous, Grade 2—thin, continuous or thick, discontinuous, and Grade 3—thick, continuous. While the subjectivity within each study may be addressed by using multiple image readers, these non-standardized methods of scoring render comparison across studies difficult.

Alternate imaging modalities such as ultrasound and MRI have also been explored (75). MRI, a more expensive imaging modality, has the additional benefit of more precisely characterizing different types of intravascular plaques (76). Although CT data is more widely available, the higher resolution afforded by MRI modalities may prove to have clinical importance in classifying plaque as being high or low risk, through the identification of intraplaque hemorrhage (IPH), a biomarker for future cerebrovascular events (15). One study found that the presence of IAC was associated with imaging markers of small vessel disease on MRI, suggesting that IAC could potentially be used as a marker for small vessel disease (33). Although preliminary studies have shown the feasibility of MRI in the accurate detection and quantification of intracranial calcification, to the best of our knowledge, currently there are no MRI-based scoring systems to quantify IAC (77, 78).

One challenge in scoring IAC lesions is differentiating between intimal and medial calcifications; while atherosclerosis of the intima is presumed to contribute to stroke the clinical significance of medial calcification is unknown. One promising new method, the Kockelkoren method, was developed specifically to differentiate intimal from medial calcification of the intracranial internal carotid artery. This method involves assigning points for circularity, thickness, and morphology (20), and was used in two of the studies we reviewed.

This method may be valuable for predicting both future stroke incidence and response to therapy, as intimal calcification is better correlated with progressive atherosclerotic lesions, and medial calcification may predict negative response to TPA but better outcomes after EVT. Although this method appears to provide clinically useful information about the histology of the lesion, it remains to be seen whether the Kockelkoren method can be used to reliably predict risk of future stroke. In summary, due to its prevalence in the clinical literature our group continues to actively use the Agatston scoring method, although we are following new literature validating the Kockelkoren method closely.

### Association of IAC With Risk of Future Stroke

Several recent prospective studies have shown a significant association between IAC and stroke risk. Two studies that prospectively followed patients after stroke or TIA for multiple years found that IAC scores were associated with higher risk of recurrent TIA (69), stroke, post-stroke mortality, and small vessel occlusive disease (70). Similarly, a case-control study specifically examining vertebrobasilar artery calcification by CT found a higher risk of recurrent stroke and vascular events with higher calcification (79).

The value of IAC as a predictor of first-ever ischemic stroke was demonstrated in the Rotterdam study. Intracranial internal carotid artery calcification volume was shown to have similar or greater predictive power than extracranial artery atherosclerosis in the prediction of future stroke among 2,323 stroke-free subjects HR 1.43 [95% CI, 1.04-1.96]) (3).

One concern is the possibility that atherosclerosis may be a confounding factor that is independently associated with both IAC (1, 80, 81) and ischemic stroke (82). The SAMMPRIS trial (Stenting vs. Aggressive Medical Therapy of Intracranial Arterial Stenosis) found that, among stenotic arteries, calcified stenosis was actually associated with a decreased likelihood of recurrent ischemic stroke compared to non-calcified stenosis (83). In fact, it has been suggested that larger calcifications might contribute to plaque stability (84). Thus, it is critical that studies seeking to establish an association between IAC and stroke account for the influence of atherosclerosis.

One way of isolating the association between calcification and stroke from confounding risk factors is using the artery contralateral to the stroke in the same patient as a control. Two studies found a higher degree of calcification ipsilateral to stroke-associated lesions. One study found that there was significantly greater calcification in the ICA ipsilateral to infarction only for stroke of undetermined etiology, not cardioembolic. In the other study, microembolic signals were more prevalent ipsilateral to internal carotid artery calcification in the high-calcium group (42, 68).

Another possible application of IAC scores may lie in the prediction of cerebrovascular response to injury. Hussein et al. found a significant association between patients with extraordinarily high calcification scores and lower rates of vasospasm after aneurysmal subarachnoid hemorrhage, suggesting a possible protective effect of arterial calcification in the context of acute brain bleeds (66).

It is important to note that the association between calcification and stroke has not gone undisputed–Taoka and colleagues 2006 found that while calcium score was positively correlated with arteriosclerotic changes in the carotid siphon and carotid bifurcation, there was no correlation between the degree of calcium and the ability to predict future strokes within a 2 year time period (61). A more recent large prospective study found no association between internal carotid artery calcification and stroke. This study included 1,349 people followed over a median follow-up of 5.1 years (72). Some of this discrepancy may come from differences in the detection and quantification of IAC. Evaluation of IAC on head CT has largely followed principles established in the CAC literature, where areas of high attenuation in arterial walls are interpreted as intra-arterial calcifications. A study by Denzel et al. showed good concordance between calcifications detected on CT and histologic analysis of carotid artery samples (41). Nevertheless, there is still no universally agreed-upon cutoff value for identifying calcification in Hounsfield units, let alone a quantitative method for IAC scoring (1). It is therefore important to consider different IAC detection and scoring methods when interpreting the results of studies of IAC and ischemic stroke risk.

Taken together, this relatively small but promising group of results suggests that IAC captured on routine CT scan may hold value in predicting recurrent stroke risk, especially among individuals with multiple risk factors.

### IAC as a Prognostic Marker for Acute Stroke Therapy

IAC may affect the outcome of therapeutic interventions for acute stroke, such as tPA or endovascular therapy (EVT). Three studies have examined the effect of IAC on tPA response. Gocmen et al. (22) conducted a retrospective study on 91 subjects who had acute anterior circulation strokes treated with IV tPA. Intracranial internal carotid artery calcification (IICAC) subtypes (medial vs. intimal) were compared to the response to IV tPA. In the study, IIIAC was diagnosed and classified according to Kockelkoren's methods. IV tPA was effective at 24 h in 48% of the subjects with no IICAC, 60% of the time in patients with intimal IICAC, and 43% of the time with medial IIAC. Medial IICAC was linked to negative early responses to IV TPA (*p* = 0.052) and increase of symptomatic intracerebral hemorrhage (21 vs. 4% intimal and no-IICAC p=0.052) (22). Similarly, in a prospective study of 982 patients, with acute ischemic stroke, IV tPA was associated with favorable clinical outcomes and recanalization in patients with medial but not intimal internal carotid artery calcification (71). Finally, Yu et al. (85) performed a retrospective study of 242 patients with acute non-cardiogenic stroke who received IV tPA and found that arterial calcification volume on the side of the lesion was associated with hemorrhagic transformation after thrombolysis. Additionally, the patients with poorer prognosis had more calcified vessels.

Compagne et al. (21) conducted a randomized prospective study on 344 subjects with acute ischemic strokes that were part of the MR CLEAN clinical trial of EVT. Intracranial internal carotid artery calcification from the horizontal part of the petrous segment to the circle of willis were measured using the Kockelkoren method. Patients with predominantly medial calcification had better functional outcome with EVT and there was no effect for EVT in patients with primarily intimal calcifications. However, there was no association between intracranial calcification volume and functional outcome (measured by 90 day modified Rankin scale) (21).

Therefore, the benefit of EVT or tPA in acute ischemic stroke patients is greater in patients with medial calcification or no calcification than in patients with intimal calcification. Compagne et al. offered the following possible explanations: either that EVT might cause greater risk of microemboli in patients with intimal calcification associated with atherosclerotic plaque, or that medial calcification may be associated with development of stronger collateral circulation pathways due to arterial stiffening.

## Discussion

IAC research presents a promising new frontier in predicting ischemic stroke risk and outcomes. Given the ubiquity of CT imaging, the addition of IAC evaluation metrics to existing imaging data could potentially enhance the identification of patients at risk for first-ever or recurrent strokes which could ultimately guide management decisions to reduce these risks. However, given the uncertainties surrounding the pathologic correlation of IAC with cerebrovascular disease, further investigation is needed to characterize the direction and magnitude of these associations. Overall, the studies reviewed here suggests that IAC identified on CT imaging may help predict recurrent stroke risk. For those individuals at highest risk, consideration may eventually be warranted for screening CT or MRI, with recommendations analogous to those applied to CAC imaging in high cardiac risk patients. However, the SAMMPRIS trial and Taoka et al. found that calcification was not positively associated with risk of future stroke. These conflicting results necessitate the development of a standardized quantitative method for IAC measurement based on imaging data. Such a method should integrate the overall IAC density as well as the distribution of IAC across different vascular territories. One important asset in this regard could be the use of deep learning, which has been established as a promising tool for predicting final infarct lesions based on day 3 imaging data (86). Another direction for further research will be to examine the predictive value of IAC in ischemic stroke treatment outcomes after medical and/or endovascular therapy. Investigation of the role of IAC in treatment outcomes could lead to precision therapy. Finally, it is also possible that IAC may predict vascular cognitive decline and overall brain health (87).

## Author contributions

GF, SR, KM, and AC are responsible for idea generation, literature search, data collection, and creating the first version of the manuscript. MS and WY are responsible for idea generation and review of the manuscript. All authors contributed to the article and approved the submitted version.

## Conflict of Interest

The authors declare that the research was conducted in the absence of any commercial or financial relationships that could be construed as a potential conflict of interest.

## Publisher's Note

All claims expressed in this article are solely those of the authors and do not necessarily represent those of their affiliated organizations, or those of the publisher, the editors and the reviewers. Any product that may be evaluated in this article, or claim that may be made by its manufacturer, is not guaranteed or endorsed by the publisher.
